# Topography and Higher Order Corneal Aberrations of the Fellow Eye in Unilateral Keratoconus

**DOI:** 10.4274/tjo.65642

**Published:** 2018-10-31

**Authors:** Mustafa Koç, Kemal Tekin

**Affiliations:** 1 Ulucanlar Eye Training and Research Hospital, Ophthalmology Clinic, Ankara, Turkey; 2 Erciş State Hospital, Ophthalmology Clinic, Van, Turkey

**Keywords:** Corneal aberrations, topography, unilateral keratoconus

## Dear Editor,

We congratulate Aksoy et al.^1^ for their study entitled “Topography and Higher Order Corneal Aberrations of the Fellow Eye in Unilateral Keratoconus”. We have read the article with interest. They evaluated and compared the topographic data and corneal higher order aberrations of fellow eyes of unilateral keratoconus patients with keratoconic eyes and control group. They retrospectively reviewed the medical records of 392 eyes of 196 patients with keratoconus and identified 20 patients (%11.2) with unilateral keratoconus. The diagnosis of unilateral keratoconus was defined as having a keratometric astigmatism below 1.5 diopter (D), vertical keratometry (K) value below 47.0 D, and no keratoconus patterns on corneal topography in this study. The results of the study revealed that there is no statistical difference in best corrected visual acuity between fellow eyes and control, whereas K_1_, K_2_, and cylindrical power values were significantly higher in the fellow eyes. Comparison of quantitative topographic indices showed that all indices except the inferior-superior ratio are significantly higher in the fellow eyes in keratoconic patients than in the control group (p<0.05). We express our gratitude to the authors regarding this study. However, we want to specify some matters and our thoughts related to this article.

First, we would like to emphasize that the term of ‘unilateral keratoconus’ is not appropriate. Because, according to the global consensus on keratoconus and ectatic diseases, keratoconus is a bilateral corneal disease.^2^ However, clinical and topographical findings of the disease may not be evident one of the eyes. Many different terms such as *subclinical keratoconus, keratoconus suspect, and forme fruste keratoconus* have been employed to describe the preclinical stages of keratoconus.^3^ We think that the term of “unilateral keratoconus” in this study may be confused with “subclinical keratoconus”. Additionally we believe that posterior corneal elevation and pachymetric index are more sensitive index for the early diagnosis of keratoconus. Hence, the patients in the study must be evaluated by using these analyses. The studies by us^4^ and Bae et al.^5^ revealed that even these analyses are not adequate to detect the subclinical keratoconus. We examined the medical records of 3474 patients with keratoconus and 116 (3.3%) cases with subclinical keratoconus were detected. The diagnosis of subclinical keratoconus was defined as having a central mean K value less than 47.2 D, an inferior-superior asymmetry for the average K less than 1.4 D, a keratoconus percentage index (KISA%) of less than 60%, and no clinical evidence. After that, these patients were analyzed with the Belin-Ambrósio Enhanced Ectasia Display (BAD) III, which evaluates the pachymetric progression and anterior and posterior elevation values of the cornea. Normal BAD analysis were detected in only 38 (1.1%) of these patients. We found that there were no statistically significant differences between the eyes with subclinical keratoconus who had normal BAD analysis and the controls in visual acuity, topographic, topometric and tomographic parameters (for all, p>0.05). We only detected statistically significant differences with regard to corneal densitometry values. Accordingly, we think that if the authors would take into account the posterior corneal surface and pachymetric indices, the prevalence of subclinical keratoconus in their study may be reduced and the keratometry values as well as some of the topographic parameters and surface index parameters might not be statistically significantly different between the fellow eyes and normal eyes.
