# Supplementary material for: Topography and Higher Order Corneal Aberrations of the Fellow Eye in Unilateral Keratoconus
Source: Turk J Ophthalmol. 2018 Oct 31;48(5):274–5. doi: 10.4274/tjo.65642 (PMC6216537; doi:10.4274/tjo.65642)
Supplement: Related texts

# Reply: Response from the Authors

**Authors:** Sibel Aksoy, Sezen Akkaya, Yelda Özkurt, Sevda Kurna, Banu Açıkalın, Tomris Şengör

## Dear Editor,

In a Letter to the Editor written in response to our article entitled “Topography and Higher Order Corneal Aberrations of the Fellow eye in Unilateral Keratoconus” published in the Turkish Journal of Ophthalmology issue 2017/5 (reference no: TJO-45220), the author claimed that the term “unilateral keratoconus” is inappropriate, and that the term ‘subclinical keratoconus’ should be used instead. The author stated that posterior elevation and pachymetry data are more sensitive indexes in the detection of subclinical keratoconus, and the use of these data would reduce the rate of subclinical keratoconus in our study.

The term unilateral keratoconus is used in the literature. These publications were also cited in our article. We stated that the eyes considered topographically and clinically normal in unilateral cases may eventually develop signs of keratoconus findings if followed long enough. The NIDEK Magellan Mapper, which is available in our clinic and was used in our study, is a Placido-based system that only provides data regarding the anterior cornea surface. The lack of posterior corneal surface data was given as one of the limitations of our study. The points criticized by the author have already been addressed in our article, as shown below:

Introduction, paragraph 2: “The progressive course of the disease ultimately affects both eyes, though only one eye may be affected initially. The prevalence of true unilateral keratoconus has been reported to range from 0.5-4% in studies using computerized videokeratography^1,2^ and was 4.5% in a more recent study using slit scanning corneal topography (Orbscan 2).^3^ Holland^2^ reported that patients with unilateral keratoconus developed keratoconus symptoms in their apparently healthy fellow eyes 4 years later, while Li et al.^4^ found that keratoconus developed in 50% of cases within 16 years. Therefore, it may be concluded that the fellow eyes of patients with unilateral keratoconus may seem normal with regard to clinical and topographical pattern but have subclinical keratoconus.”

Discussion, last paragraph: “In our study, the unilateral keratoconus ratio was found to be 11.2%. The prevalence of true unilateral keratoconus is reported in the international literature as ranging between 0.5% and 4.5%. In a study conducted in Turkey, a unilateral keratoconus prevalence of 14.9% was determined using Pentacam.^5^ The main limitation of our study was that the elevation data provided by our topography device was not adequate and not able to evaluate the posterior corneal surface. Another limitation is that long-term patient follow-up data was not available. The suspected keratoconus eyes that we determined to be normal may exhibit signs that would lead to a keratoconus diagnosis if examined using more advanced topography systems.”

Best Regards,

## References

[1] Rabinowitz YS, Nesburn AB, McDonnell PJ (1993). Videokeratography of the fellow eye in unilateral keratoconus. Ophthalmology..

[2] Holland DR, Maeda N, Hannush SB, Riveroll LH, Green MT, Klyce SD, Wilson SE (1997). Unilateral keratoconus; incidence and quantitative topographic analysis. Ophthalmology..

[3] Wei RH, Zhao SZ, Lim L, Tan DT (2011). Incidence and characteristics of unilateral keratoconus classified on corneal topography. J Refract Surg..

[4] Li X, Rabinowitz YS, Rasheed K, Yang H (2004). Longitudinal study of the normal eyes in unilateral keratoconus patients. Ophthalmology..

[5] Oruçoğlu F (2013). Unilateral keratokonuslarda insidans ve tomografik değerlendirme. Turk J Ophthalmol..
